# Supplementary material for: Temporal trends in Plasmodium vivax diversity in eastern Cambodia evidence declining transmission
Source: medRxiv. 2026 Mar 4:2026.03.03.26346840. Preprint. [Version 1] doi: 10.64898/2026.03.03.26346840 (PMC13004073; doi:10.64898/2026.03.03.26346840)
Supplement: Supplement 1 [file media-1.pdf]

Supplementary Information for:

Temporal trends in *Plasmodium vivax* diversity in eastern Cambodia evidence declining transmission

Rotha Eam[1], Kian Soon Hoon[2], Edwin Sutanto[2], Anjana Rai[2], Hidayat Trimarsanto[2], Angela Rumaseb[2], Sopheany Thin[1], Sreyneat Hor[1], Chhea Chhorvann[3], Tol Bunkea[4], Ric N Price[2,5,6], Jean Popovici[1,7*], Sarah Auburn[2,5,6*]

1 Malaria Research Unit, Institut Pasteur du Cambodge, Phnom Penh, Cambodia
2 Global and Tropical Health Division, Menzies School of Health Research and Charles Darwin University, Northern Territory, Australia
3 National Institute of Public Health, School of Public Health, Phnom Penh, Cambodia
4 National Center for Malaria Control, Phnom Penh, Cambodia
5 Mahidol-Oxford Tropical Medicine Research Unit, Mahidol University, Thailand
6 Centre for Tropical Medicine and Global Health, Nuffield Department of Medicine, University of Oxford, Oxford, United Kingdom
7 Infectious Disease Epidemiology and Analytics G5 Unit, Institut Pasteur, Paris, France

*These authors made equal contributions. Address correspondence to: jpopovici@pasteur-kh.org or sarah.auburn@menzies.edu.au

Period (years)	Province	Sex		Age (median, IQR)	Treatment	Number of samples
		**Male**	**Female**			
**2014**	Rattanakiri	65% (24/37)	35% (13/37)	20 (4-60)	DHA-PPQ	37
**2015**	Rattanakiri	73% (33/45)	27% (12/45)	25 (8-60)	AS-MQ	45
**2019**	Mondulkiri	96% (48/50)	4% (2/50)	27 (15-57)	AS-MQ	50
**2023**	Mondulkiri	65% (32/49)	35%(17/49)	19 (9-54)	AS-MQ + PQ	49

Supplementary Table 1. Socio-demographic details from retrospective data of symptomatic *Plasmodium vivax* infections. *Total number of *P. vivax* cases reported in the district. NA, not applicable. Case reports were not available in 2014. DHA: dihydroartemisinin, PPQ: piperaquine, AS: artesunate, MQ: mefloquine, PQ: primaquine

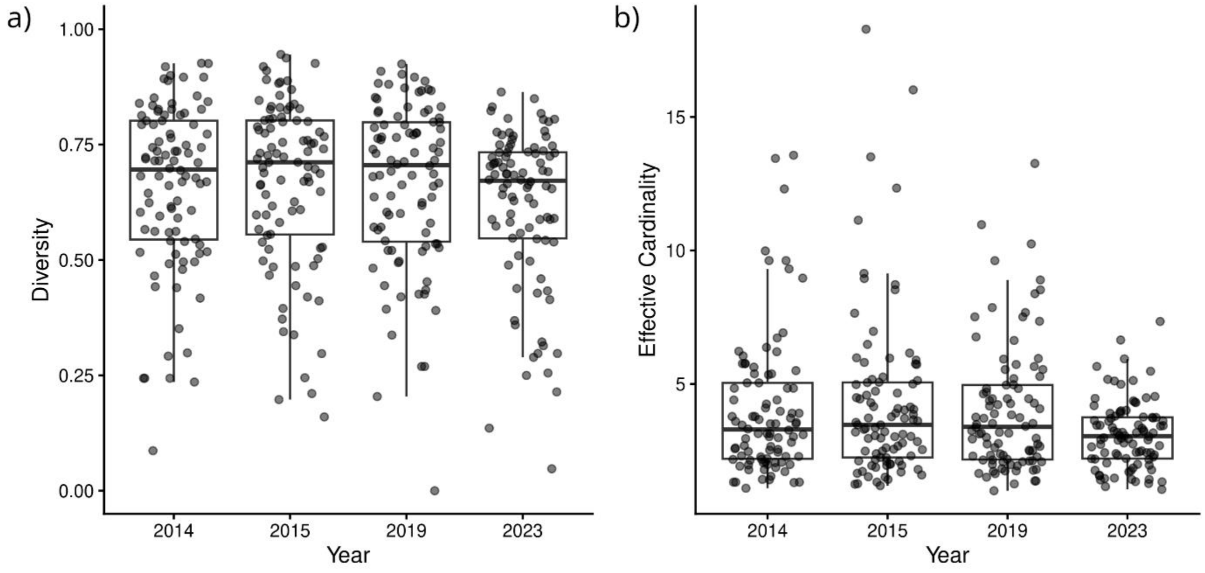

Supplementary Figure 1. Marker-based allele diversity and effective cardinality in each of the study years. Data is presented on the 92 high performance microhaplotype markers in the 110 monoclonal samples.

Period (year)	% polyclonal cases	Mean eMOI*	Mean population HE**	Mean marker HE**	Mean marker Eff Card***
2014	41% (15/37)	1.26	0.059	0.66	4.02
2015	22% (10/45)	1.19	0.051	0.67	4.26
2019	48% (24/50)	1.42	0.074	0.66	4.03
2023	4% (2/43)	1.03	0.01	0.62	3.11

Supplementary Table 2. Summary statistics on within-host, population-level and marker diversity. *eMOI, effective multiplicity of infection. **HE, expected heterozygosity. ***Eff Card, effective cardinality.

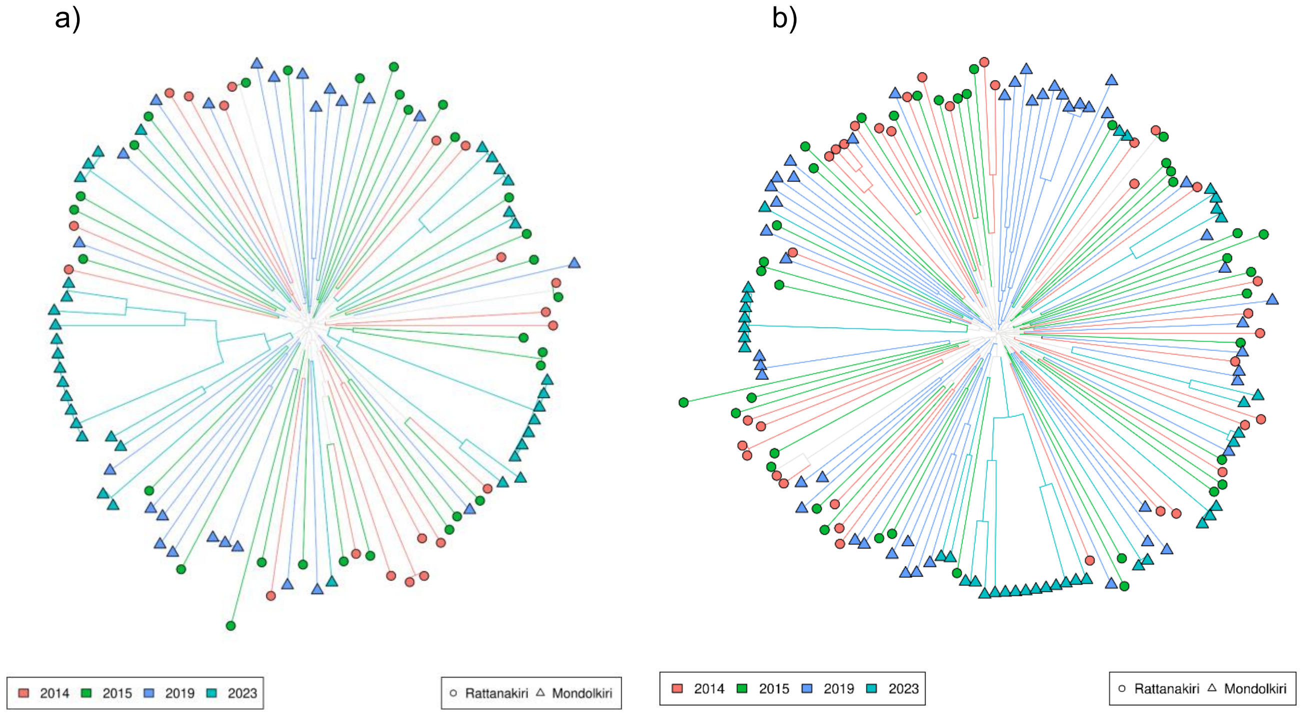

Supplementary Figure 2. Intermixing of infections across the provinces and years of collection. Panel a) presents a neighbor-joining tree generated on 110 monoclonal samples using 86 microhaplotype markers with no missing data in the sample set. Panel b) presents a neighbour-joining tree generated using data on 159 samples (monoclonal and polyclonal) with 86 microhaplotype markers. Major allele calls were used for heterozygote positions in the polyclonal infections.
